# Extreme Value Theory Applications to Space Radiation Damage Assessment in Satellite Microelectronics

**DOI:** 10.6028/jres.099.046

**Published:** 1994

**Authors:** P. W. Marshall, C. J. Dale, E. A. Burke

**Affiliations:** Naval Research Laboratory, Washington, DC 20375 and SFA, Inc., Landover, MD 20785; SFA, Inc., Landover, MD 20785; Woburn, MA 01801

**Keywords:** charge coupled devices, charge injection devices, displacement damage, extreme value statistics, microdosimetry, satellite imagery, satellite microelectronics

## Abstract

Calculations of the first and second moments of displacement damage energy distributions from clastic collisions and from nuclear reactions, at proton energies ranging from 10 MeV to 300 MeV, are incorporated into a model describing the probability of damage as a function of the proton fluence and the size of the sensitive micro-volume in Si. Comparisons between the predicted and measured leakage currents in Si imaging arrays illustrate how the Poisson distribution of higher energy nuclear reaction recoils affects the pixel-to-pixel variance in the damage across the array for proton exposures equivalent to mission duration of a few years within the earth’s trapped proton belts. Extreme value statistics (EVS) quantify the largest expected damage extremes following a given proton fluence, and an analysis derived from the first-principle damage calculations shows excellent agreement with the measured extremes. EVS is also used to demonstrate the presence of high dark current pixels, or “spikes,” which occur from different mechanisms. Different sources of spikes were seen in two different imager designs.

## 1. Introduction

Proton-induced displacement damage degrades semiconductor electrical properties by introducing localized energy states within the band-gap which result in increased generation dark current, carrier recombination and charge trapping. On *average*, the permanent proton-induced damage in bulk Si is proportional to the average amount of energy which has been imparted through non-ionizing processes following elastic and inelastic scattering of Si atoms [1–3]. However, on micro-volume scales appropriate for microelectronics, average damage is a poor indicator of damage effects because of differences in the number of incident particles and fluctuations in energy deposition which are an unavoidable consequence of collision kinematics.

Characterization of displacement damage in Si micro-volumes has particular importance for satellite imaging array applications. Device radiation hardening solutions have largely solved problems associated with ionization effects. However, particle irradiation seriously degrades charge transfer efficiency through carrier trapping and increases dark current by carrier generation. Permanent dark current increases from single particle interactions have been reported in sensor arrays following proton and neutron irradiation [4,5]. Pixel-to-pixel variations in dark current increases following multiple interactions within each pixel have also been shown to depend on the incident particle and energy [3,6].

Orbital proton energy spectra, whether from the earth’s trapped radiation belts or solar flares, typically peak at very low (and more damaging) energies and decrease exponentially with increasing proton energy. Typical spacecraft structural shielding effectively attenuates lower energy protons resulting in spectra extending from a few MeV to several hundred MeV with average energies over 20 MeV. Proton linear accelerators and cyclotrons are therefore well suited for monoencrgetic characterizations of damage verses proton energy which can then be incorporated in damage predictions for a given environment and shielding configuration.

For the proton energy range of 10 MeV to 300 MeV, this work explains the average damage and pixel-to-pixel damage fluctuations in terms of calculated parameters reflecting the energy dependence of the proton-silicon interactions. The analysis predicts the damage distribution within a given array as illustrated for the particular case of a charge injection device (CID) depletion volume and the cross-sections and Si recoil energies applicable to 12 MeV, 22 MeV, and 63 MeV proton induced damage. This enables a direct comparison between the predicted damage distributions and the observed dark current histograms reported in [3] across a range of energies important for orbital environments.

Dark current extremes, which may follow from damage extremes, are a particularly serious concern for a variety of satellite imager applications. These “hot pixels” of “spikes” interfere with the instruments ability to resolve small, dim objects such as low magnitude stars which might be used for a star tracker guidance system. Also, spikes in a image can place overhead on data compression algorithms and burden telemetry channels. Extreme value statistics are well suited for characterizing the frequency and magnitude of these spikes. These tools are applied to proton damaged CID imagers to illustrate this approach, and we show that for one particular CID design, the spikes can be accurately predicted based on the calculated probabilities and kinematics of proton-initiated nuclear reactions.

## 2. Recoil Spectra Parameters

**Fig. 1:**
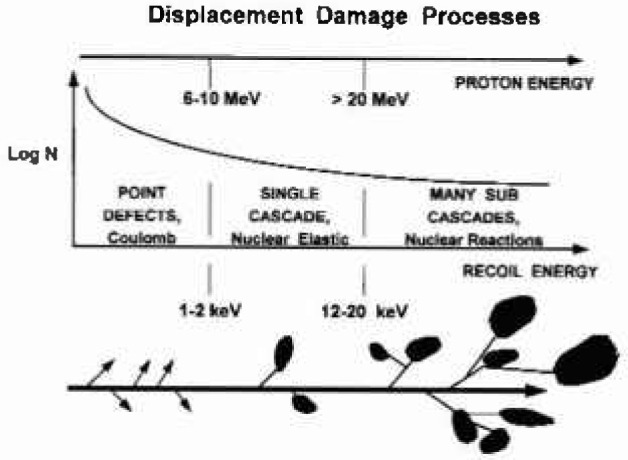
Frequent coulombic scattering from protoni of a few McV initiate low energy recoil atoms resulting in isolated defect sites. More energetic protons can impart more energy to recoil atoms via nuclear elastic and inelastic reactions resulting in less frequent but more complex damage structures.

For proton energies of practical interest in satellite orbits, the damage is caused by recoiling atoms from collisions with Si atoms. As depicted in Fig. 1 [7], elastic scattering by the Coulombic field of the nucleus dominates for protons below 10 MeV, though at higher energies, nuclear elastic scattering also becomes important. By 60 MeV, about half of the displacement damage is due to nuclear inelastic reactions which dominate above 100 MeV. Elastic cross-sections are relatively high with recoil energies typically less than 1 keV as opposed to infrequent nuclear reactions emitting very damaging MeV-range recoils. In this work, the first and second moments of the recoil spectra are calculated separately for each type of interaction.

**Fig. 2:**
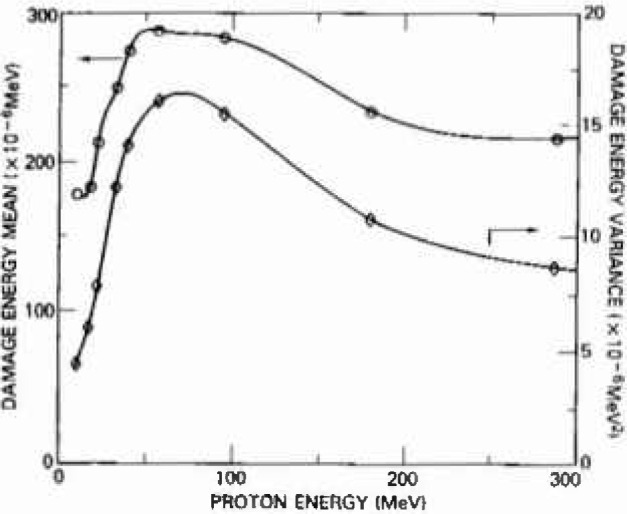
The mean and variance of the local elastic damage energy arc plotted versus proton energy along with a best-fit curve. The moments were calculated based on clastic differential cross-section data [1 and references therein] indicated by circles and triangles.

The average damage energy from all *elastic* recoils is obtained by numerically integrating the product of the differential cross section weighted by the corresponding recoil damage energy, over all scattering angles. Damage energy is defined here as the portion of energy lost by a recoil through mechanisms other than ionization as calculated by Lindhard et al. [8]. Note that this represents an important adjustment to the total energy imparted by the reaction atoms which must be assessed for evaluating either the nonionizing or the ionizing energy imparted. The second moment calculations proceed in the same manner, except the recoil damage energies now appear to the second power. The variance follows as the second moment minus the first moment squared, as is customary. Figure 2 plots the mean and variance of non-ionizing energy for proton energies from 10 MeV to 300 MeV, and Table 1 lists the values of experimental interest, along with the total elastic cross-sections and recoil energies.

The *inelastic* reaction cross-sections are estimated according to the empirical formula of Letaw et al. [9]. Calculations of primary recoil energies consider both the initial intranuclear cascade and subsequent evaporation of nucleons. The momentum imparted during the evaporation phase is estimated using a Brownian motion model. Next, the average and variance of the damage energy are calculated as in the elastic case, and the results are summarized in Table 1. Further details and comparisons with data are discussed in [1,3].

## 3. Damage Calculations

For a given proton energy, the mean and variance describing the probability density function (pdf) for damage from single interactions, as listed in Table 1, allow independent evaluation of the damage expected from the elastic and inelastic recoil categories. For the *elastic* category the mean for the pdf describing damage at a given proton fluence is the product of the number of interactions and the mean of the pdf for single interactions. The number of interactions is the product of the average cross-section, the incident particle fluence, and the number of Si atoms in the sensitive volume.

The elastic scattering component of the variance associated with the fluence dependent pdf is estimated as the product of the number of interactions and the single interaction pdf variance shown in Fig. 2. This is possible because Poisson fluctuations in the number of elastic recoils per pixel do not contribute significantly to the final result. In the regime where *N*, the average number of interactions per volume element, is greater than 20, the *N*-fold convolution of the single interaction pdf with itself leads to a Gaussian elastic damage distribution with mean and variance as described above.

For sensitive volumes and fluences of interest here, the average number of *inelastic* recoils ranges typically from a fraction to a few, and a discrete Poisson distribution determines the probability of a given number of inelastic recoils. The pdf governing the inelastic damage energy for a pixel with *N_i_* inelastic recoils reflects the *N_i_*-fold convolution of the pdf for single inelastic damage. For purposes of this analysis, the form of the single event pdf for inelastic recoil products is approximated as a two parameter gamma distribution with mean and variance as indicated in Table 1.

**Fig. 3:**
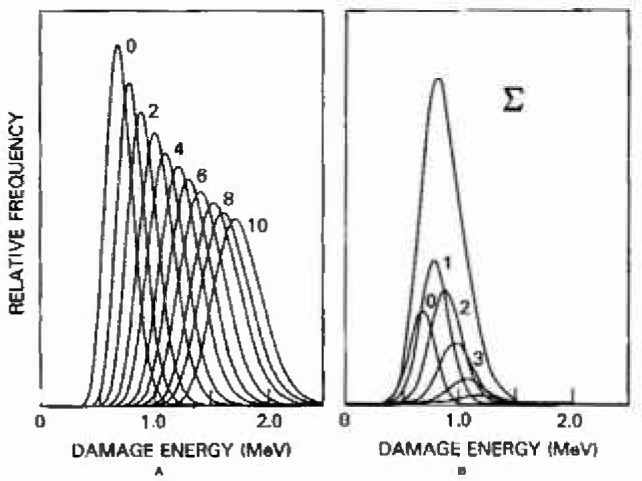
(a). The Gaussian distribution with no inelastic recoils describes elastic damage and convolved distributions show combined damage from clastic and 1 through 10 inclusile recoils. (b) Weighting according to the Poisson probabilities precedes the superposition to determine combined damage probabilities. The simulation applies to the Si CID sensitive volume of 1300 μm^3^ and 4.0 × 10^10^ 12 MeV protons/cm^2^.

Since the elastic and inelastic processes are independent random variables, the *combined* damage for pixels in which both occur follows as the convolution of the pdfs describing each of the two categories. Figure 3 illustrates this simulation for the specific case of the imaging array used in this study in which damage from a fluence of 4.0 × 10^10^ 12 MeV protons/cm^2^ occurs, and each pixel’s sensitive volume is 1300 μm^3^. The Gaussian distribution, shown in Fig. 3a as the *N_i_* = 0 case, describes damage corresponding to an average of 4,000 events per pixel. Figure 3a curves for *N_i_* = 1 through 10 inelastic recoils per pixel reflect increases in both the means and variances as the shape tends toward Gaussian. Figure 3b shows the pdf for total combined damage as the superposition of the pdfs in Fig. 3a, after weighting by their associated Poisson probabilities according to the average of 1.8 inelastic recoils per pixel. This average is arrived at by considering the number of silicon atoms present in the 1300 μm^3^ volume, and the composite cross-section for nuclear inelastic reactions for 12 MeV protons as shown in Table 1.

Early in a space mission or in a relatively benign orbit, the fluences may be 1–2 orders of magnitude lower, at about 10^8^ cm^−2^. In this low fluence regime, the very low probability of inelastic recoils suggests that two would probably not be observed in the same volume element. The number of elastic recoils per volume would be correspondingly low resulting in very large relative changes within the pixels where nuclear reactions occur. The product of the low probability of an inelastic event with the large number of pixels determines the pixel population for which damage exceeds the average by factors of up to 1,000.

## 4. Predicting Damage Extremes

In addition to being a necessary tool for assessing radiation-induced fixed pattern noise, the probability density function describing damage throughout the array can be used to predict the number of elements sustaining exceedingly large damage increases after a specified exposure. In [6] it was shown how extreme value statistical analysis can describe the measured distribution of pixels with the largest damage increases following 12 MeV and 63 MeV proton damage to the Si CID. For a broad range of proton energies and fluence levels, the largest extremes were shown to obey a Type 1 extreme value distribution. Next it will be shown that the particular Type 1 distribution describing proton-induced damage extremes can be predicted from the calculated pdf described above.

**Fig. 4:**
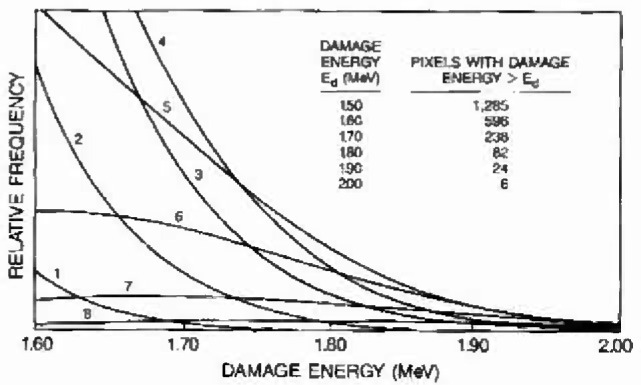
For the simulation depicted in Fig. 3, volumes containing from 1 to 10 inclastic recoils contribuite to the population of pixels with the most damage. The inset shows the number expected above a given damage energy for a 61,504 pixel array.

Figure 4 shows an expanded view of the tail region in Fig. 3b which identifies the contributions to the pdf from the 11 populations containing 0 through 10 inelastic recoil events per pixel. The damage energy distribution has a mean of 0.85 MeV, and the skewed high energy tail extends to about 1.8 MeV. Individual distributions are identified according to the number of inelastic recoils. Figure 4 illustrates how several of the component distributions contribute to the probability of exceeding large damage energies. Based on a total pixel population of 61,504, the inset presents the number of pixels expected above the specified damage level, *E_d_.* This number is the total population multiplied by *p*_d_, the probability of exceeding damage energy *E*_d_ within the whole array. This probability is calculated as the summed pdf integrated from *E*_d_ to infinity.

Two steps are necessary to compare these results on the basis of the cumulative Type 1 extreme value distribution. As discussed in [6,10–12], extreme value analysis can be applied to data to evaluate the probability of exceeding a certain value within any population size by evaluating a set of largest values extracted from subsets of a given population. In the next section we will treat the case where the 61,504 pixel population has been subdivided into 248 groups of 248 pixels each. Using *p*_d_ as defined above, the probability, *p*_0_, of having no pixels exceeding *E*_d_ within the group of 248 pixels can be evaluated using the discrete binomial distribution as:

$$p_{0}={\left(1-p_{d}\right)}^{248}.$$ (1)

The associated standard variate specific to the extreme value cumulative probability plot is given by:

$$S\left(p_{0}\right)=-\ln\left[-\ln\left(p_{0}\right)\right].$$ (2)

Thus *E*_d_, or a proportional quantity such as dark current, can be plotted against the corresponding standard variate to predict the Type 1 extreme distribution specific to the pdf from which it is generated. Detailed discussions of extreme value analysis are discussed in the references [11,12], and applications to this study will be illustrated in the following section.

## 5. Comparison with Dark Current Data

Calculations described in the previous section are compared here to measured dark current increase distributions specific to proton-induced damage in a General Electric 256 pixel × 256 pixel Si CID. Devices are fabricated in an n-type Si epitaxial layer doped with 5 × 10^14^ P atoms/cm^2^. A field isolation oxide confines the collection area to about 17 mm × 17 mm, but for purposes of dark current studies only the 1300 µm^3^ depletion volume leads to carrier generation.

All dark current data reported here were acquired at 18.0 °C and correspond to a 248 × 248 subset of the array. After each proton exposure and measurement the dark current increase for each element was calculated by a pixel-by-pixel subtraction of the pre-irradiation value. This correlation removes imager spatial noise not resulting from radiation. Temporal read-out noise accounts for less than 5% of the dark current spreads reported here. More detailed descriptions of the imaging array and the dark current measurement are provided in [6].

Proton irradiations with energies of 12 MeV, 22 MeV, and 63 MeV were performed at the University of California at Davis cyclotron facility. The beam line and dosimetry have been described previously [13]. Irradiations were conducted at a nominal dose rate of 1 kRad(Si)/s with all leads grounded. Dark current measurements were initiated about 15 minutes post irradiation and repeated after 1 day and again after about 1 week. No significant annealing was observed over this period.

**Fig. 5:**
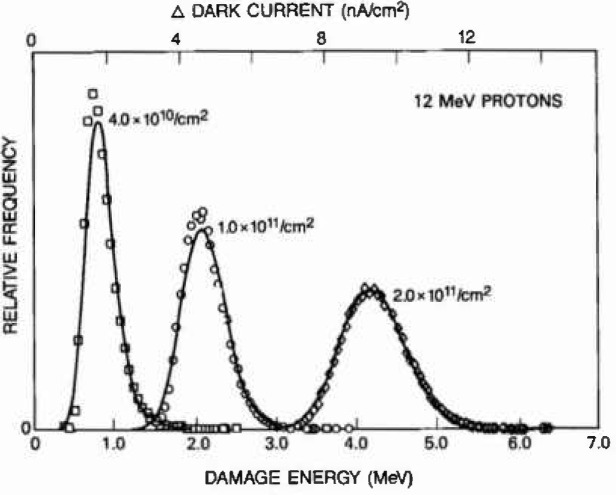
Calculated damage energy distributions show excellent agreement with measured dark current histograms from a Si CID damaged by 12 MeV protons. Calculations are based un averages of (1.8, 4.5, and 9.0) inelastic recoils per pixel, and the damage distribution shapes reflect the associated discrete Poisson distributions.

In Fig. 5, comparisons are made between dark current data histograms and calculated damage energy distributions in the CID pixels. The calculation approach described above has been exercised for three 12 MeV proton fluences corresponding to averages of (1.8, 4.5, and 9.0) inelastic recoils/pixel. Based on the population of 61,504 pixels and Poisson statistics, the maximum numbers of inelastic recoils expected in any single pixel are 10, 16, and 24, respectively. For comparing the calculations to dark current data, a conversion factor relates the average dark current and the mean damage energy. For the three fluences, the average conversion factor of 2.2 nA/cm^2^ per MeV of damage energy varies by up to 10%, which reflects the experiment’s dosimetry uncertainty. The calculated damage curves in Fig. 5, based on the first and second moments of the non-ionizing energy imparted by the recoil spectrum, describe the dark current distribution to a remarkable degree of accuracy.

Comparisons for 22 MeV and 63 MeV proton damage show similar agreement. The coefficient of variation, defined as the ratio of the standard deviation to the mean damage (or dark current), is a dimensionless figure-of-merit. At 12 MeV, 22 MeV, and 63 MeV, the experimental and calculated results agreed within 2%, 12%, and 15% respectively [14]. Also at 63 MeV, with 45% of the damage caused by inelastic recoils, the means of the two distributions are normalized by a factor of 2.0 nA/cm^2^ per MeV of damage. This does not differ significantly from the conversion factors determined for 12 MeV, thus demonstrating that the average damage is proportional to the energy lost through non-ionizing processes, and that the expected damage from both the elastic and inelastic categories is present.

The somewhat better agreement between calculated and measured damage distributions at the lower proton energy of 12 MeV could be influenced by characteristics associated with high energy recoils. At proton energies of 12 MeV and 63 MeV, the contribution to the total damage from inelastic reaction recoils increases from roughly 15% to 45%. Also, as this fraction increases, the average inelastic recoil energy (and range) also increases, and at higher proton energies the higher energy recoil ranges approach the smallest dimension of the sensitive volume (about 2 µm). These issues would be even more important for smaller sensitive volumes (i.e., CTE loss in a CCDs buried channel).

## 6. Largest Dark Current Extremes

**Fig. 6:**
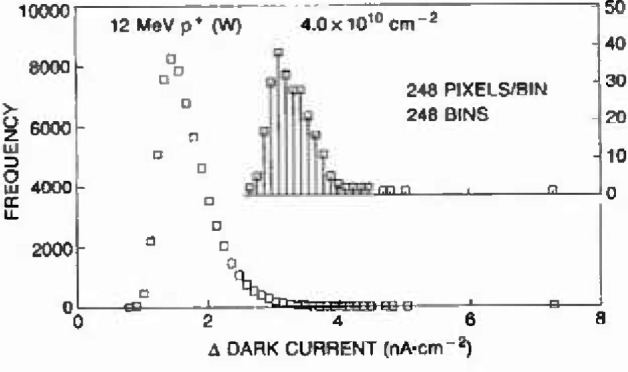
Measured dark current histogram for 61,504 pixels following exposure to 4.0 × 10^10^ 12 MeV protons/cm^2^. The 248 extremes are from groups of 248 pixels.

Here the measured largest dark current increases are compared to the calculated damage maxima for the specific cases of the three 12 MeV proton fluences of Fig. 5. For each proton energy and fluence level, the dark current extreme populations are generated by subdividing the 61,504 pixel population into 248 groups of 248 pixels each. The largest value from each group comprises the population of extremes. Figure 6 depicts how the extreme distribution is derived for the case of the lowest fluence level shown in Fig. 5 (note this example also corresponds to the calculations for Figs. 3b and 4). After ranking and estimating the probability according to the [rank/(*n* + 1)] for *n* samples as in [6], the standard variate follows from Eq. (2), and the measured dark current extremes can be compared with the Type 1 extreme value distribution using a Type 1 cumulative probability chart.

**Fig. 7:**
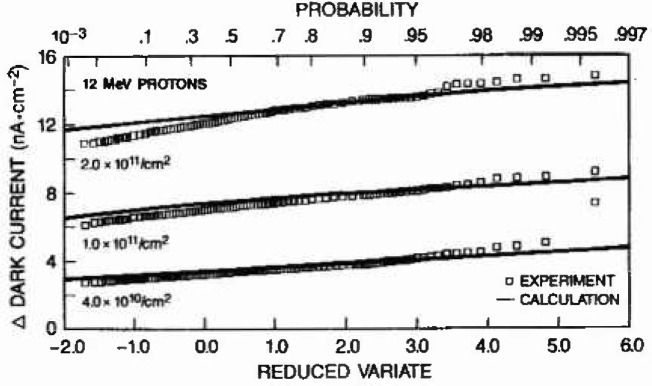
Cumulative probability distributions demonstrate excellent agreement between calculated damage extremes and the measured dark current extremes based on a 248 pixel by 248 group extreme value analysis. Though not shown here, similar agreement is obtained fur damage from 63 MeV protons.

Likewise, damage maxima calculated as described in section C can be compared to the same Type 1 extreme probability distribution using Eqs. (1 and 2) and the normalization constant of 2.2 nA/cm^2^ per MeV of damage energy. Figure 7 compares measured dark current extremes, for the three 12 MeV proton fluences treated in Fig. 5, to predicted damage maxima according to the Type 1 distribution. The linear character of the data and calculation show that they obey a Type 1 distribution, and the close agreement at each fluence demonstrates the robustness of the analysis. The return period abscissa at the top of Fig. 7 identifies the largest expected dark current increase for a given number of array subsets. For example, at the fluence of 2.0 × 10^11^/cm^2^ the return period value of 10 corresponds to about 13 nA/cm^2^ indicating the largest expected increase within a set of 10 groups or 2,480 pixels. Good agreement also exists between the measured and predicted extremes from 63 MeV protons.

The ability of the calculation to predict the largest measured dark current changes offers insight into the mechanisms responsible for proton-induced damage extremes. The linear response on the Type 1 plot indicates that a single mechanism is probably responsible for largest values while the slope reflects the variance. As pointed out in Fig. 4, the largest damage regions in this fluence regime follow from the probabilistic treatment of pixel populations damaged by several inelastic reaction recoils.

When the probability of an inelastic recoil per pixel is much less than one, as is the case in many natural space environments, the analysis can determine the total number of pixels expected with damage above a given level. In this regime, where the background radiation-induced damage can be quite low, largest damage regions can be several hundred times the average. Some of the array subsets would have largest changes dominated by single inelastic recoil damage and others by the largest of the less damaging elastic recoils. In this case, agreement with the Type I cumulative chart could be expected only with sufficiently larger bin sizes so that each bin would include at least 1 pixel with damage from an inelastic reaction.

**Fig. 8:**
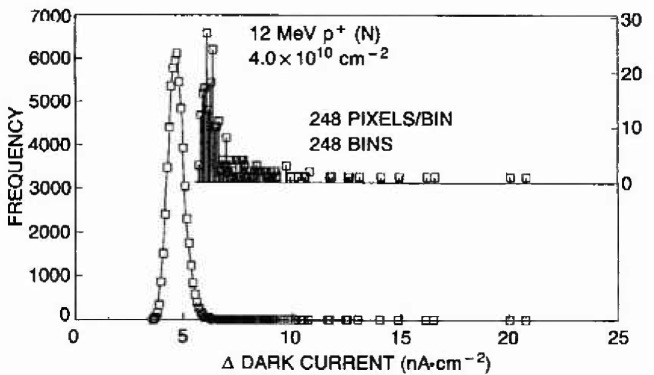
The high electric field CID design yields a different dark current and extreme response as Compared to the same conditions shown in Fig. 6. High electric fields are thought enhance the leakage currents when associated with damage.

**Fig. 9:**
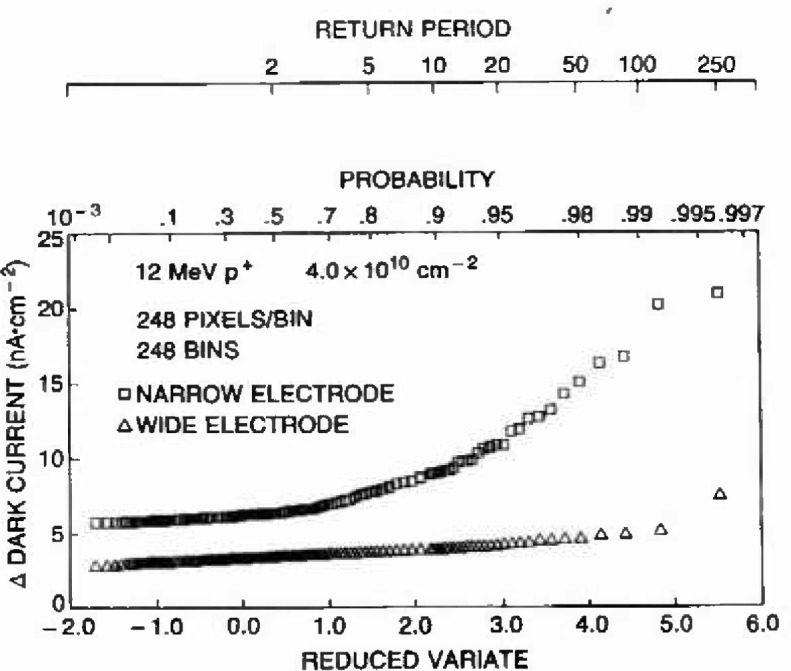
The probability chart comparing the responses shown in Figs. 6 and 8 suggests the role of field enhanced mechanism in causing the largest leakage extremes.

A qualitative comparison of such a situation follows from our evaluation of the proton response of an alternate CID imager design. The important aspects of this “narrow row” design were previously discussed in [6], with the key difference resulting in spurious high electric field profiles near the row electrodes. The comparison of the dark current and extreme distributions for this device type, shown in Fig. 8, can be made with the previously discussed design at the same proton exposure level, as in Fig. 6. Note that the average dark current is doubled, but more importantly, the character of the extreme distribution is markedly different. The consequence of this is more evident in the probability chart of Fig. 9. Clearly the narrow row design results in an extreme distribution which is not Type 1 when analyzed as before. Rearrangement of the array to 31 bins of 1984 pixels offered a better match with the Type 1 distribution. Even so, the extremes for this case cannot be understood based on first principles analysis of damage mechanisms as before. We concluded that in this case, the largest extremes were not caused by conventional charge generation, and extreme value statistics played a critical role toward quantifying the likelihood and magnitudes of this other mechanism. In [4] we discuss supplemental measurements and analysis which have lead us to conclude that the high field regions were causing localized lowering of the band-gap resulting in field enhanced emission and tunneling currents. Thus the statistics of extremes are applied to evaluate design variations and to assure that optimum imager performance can be assured. We also concluded that acceptable designs should have extreme characteristics as depicted in Fig. 6 which are limited only by unavoidable consequences of particle-semiconductor physics.

## 7. Conclusions

This paper presents an analytic approach for determining the pixel-to-pixel distribution of particle-induced displacement damage in micro-volumes representing sensitive volumes in sensor arrays. The calculation is based on interaction cross-sections as well as parameters describing the damage imparted by the spectrum of particle-initiated recoils. It predicts the dark current distribution and largest dark current changes in a Si CID following incremental damage with 12 MeV, 22 MeV, and 63 MeV protons. These proton energies span a regime important to the natural space environment; lower energy protons for which the damage is dominated by elastic scattering and higher energies where nuclear reactions become increasingly important. The analysis illustrates how high energy recoils from nuclear reactions influence the pixel-to-pixel variance in proton-induced damage and cause the largest damage occurrences. To understand the important exception, we rely on extreme value statistics to identify and quantify the role of electric field enhanced emission as a mechanism for excessive leakage currents.

The calculation is general in the sense that once the parameters describing the recoil spectrum are determined, the particle-induced damage distribution can be calculated as a function of particle type, particle fluence, sensitive volume, and material. The significance of these results is that once the factor relating the mean dark current to the damage energy is known from a single measurement on a particular array, the radiation response in a specified environment can be predicted. In addition to providing a means for assessing the radiation response of a given imager, this analysis has flexibility enabling the design-phase evaluation of the radiation response of different pixel geometry and materials in a variety of environments.

Extreme value statistics play a critically important role in understanding leakage current spikes and in assuring reliable satellite performance. In ongoing related research we continue to rely on this valuable tool for assessing damage and single particle ionization extremes in infrared imaging arrays and in optoelectronic detector materials for high data rate spacecraft data links, each of which must perform to exacting standards to assure reliable performance of extremely valuable space assets.

## Figures and Tables

**Table 1:** Proton recoil spectrum parameters

Proton energy (MeV)	Cross section (BARNS)	Mean recoil energy (MeV)	Mean damage energy (MeV)	Variance of damage energy (MeV)^2^
Elastic reactions				

t2	1548	3.40 × 10^−4^	1.76 × 10^−4^	4.77 × 10^−6^
22	857	4.63 × 10^−4^	2.13 × 10^−4^	7.71 × 10^−6^
63	318	7.77 × 10^−4^	2.87 × 10^−4^	1.62 × 10^−5^

Inelastic reactions				

12	0.670	0.267	0.0765	2.05 × 10^−3^
22	0.723	0.569	0.111	2.71 × 10^−3^
63	0.523	1.44	0.152	3.11 × 10^−3^
